# Establishment of Genetic Transformation System of Non-Embryogenic Callus in *Rosa rugosa*

**DOI:** 10.3390/cimb47110894

**Published:** 2025-10-28

**Authors:** Xinyun Liu, Xiyang Zhu, Yating Yang, Guo Wei, Liguo Feng, Mengjuan Bai

**Affiliations:** College of Horticulture and Landscape Architecture, Yangzhou University, Yangzhou 225009, China; 18961528363@163.com (X.L.); 13915849056@163.com (X.Z.); mx120230858@stu.yzu.edu.cn (Y.Y.); gwei@yzu.edu.cn (G.W.); lgfeng@yzu.edu.cn (L.F.)

**Keywords:** callus, genetic transformation, *RrPSY1*, *Rosa rugosa*

## Abstract

*Rosa rugosa* (*R. rugosa*) is a commercially important ornamental species within the genus Rosa, highly valued in the horticultural market. With the increasing availability and improved annotation of Rosa genomes, establishing an efficient genetic transformation system has become essential for validating candidate gene functions. As a common intermediate tissue in plant regeneration, callus has been successfully used to establish genetic transformation systems in numerous species. In this study, we characterized the morphological and physiological differences between embryogenic and non-embryogenic calli in *R. rugosa*. The embryogenic callus exhibited significantly higher catalase (CAT) activity and proline (PRO) content than the non-embryogenic callus. However, its growth rate was markedly slower. Antibiotic sensitivity assays identified the optimal selection concentrations for non-embryogenic callus as 35 mg/L for kanamycin and 13 mg/L for hygromycin. We subsequently introduced the *phytoene synthase* (*RrPSY1*) gene into non-embryogenic callus, with positive transformants identified using GFP fluorescence detection and PCR analysis. The overexpression of *RrPSY1* significantly increased the yellow pigment substances in the callus, confirming the establishment of an effective genetic transformation system for non-embryogenic calli in *R. rugosa.* This system provides a useful technical platform for the manipulation of metabolic products and the verification of related gene functions in rose.

## 1. Introduction

Somatic embryogenesis represents a vital pathway for plant regeneration and serves as a foundational step in establishing genetic transformation systems [1]. Somatic embryogenesis can be induced in vitro via direct or indirect pathways. The direct pathway leads to embryo formation on tissue surfaces, while the indirect pathway involves a callus intermediate. A significant challenge in the indirect pathway is the inevitable concomitant development of non-embryogenic callus [2]. Furthermore, during prolonged subculture, embryogenic callus is susceptible to browning, cell death, senescence, and a potential loss of embryogenic capacity, ultimately leading to its conversion into non-embryogenic callus [3,4,5].

Over the past few decades, multifaceted comparisons between embryogenic and non-embryogenic calli across various plant species have revealed distinct morphological, physiological, and biochemical differences. Generally, embryogenic callus exhibits a more compact structure and higher levels of beneficial metabolites, including soluble sugars, soluble proteins, and proline, which are believed to support its regeneration capacity [6,7,8,9,10]. Recent transcriptomic, proteomic, and epigenetic studies have further elucidated the molecular basis of these differences, showing that differentially expressed genes are predominantly enriched in pathways related to hormone signaling, sugar transport, stress response, and metabolism [11,12,13].

Although non-embryogenic calli lack the capacity for plant regeneration, they possess distinct advantages for biotechnological applications, including rapid proliferation, year-round availability free from seasonal or environmental constraints, and ease of harvest [14]. In addition, the absence of complex developmental processes in non-embryogenic callus makes it particularly suitable for gene functional studies that do not require whole-plant phenotypic observation [15,16]. These qualities make them ideal systems for both the production of secondary metabolites and the verification of gene functions. For instance, in citrus, callus systems have been utilized to demonstrate that genes such as *CrWRKY42*, *CsMADS5* and *CitTRL* are involved in regulating the synthesis of chlorophyll, anthocyanins, and carotenoids [17,18,19]. Apple callus has been utilized to confirm that *MdMYB10* and *MdMYB9* enhance anthocyanin synthesis, while *MdPSY2-1* promotes the production of total carotenoids [20,21]. Overexpression of *PSY* in peach callus significantly increased the content of carotene [22]. Moreover, the callus-based genetic transformation system has been widely adopted for functional validation of stress-responsive genes, owing to its distinct advantages such as extended experimental timelines and high-throughput capability [13,19,23,24].

*Rosa rugosa* Thunb., an upright deciduous shrub within the Rosa genus, is native to East Asia and was introduced to Europe and North America in the mid-19th century [25,26]. Valued for its vibrant blooms, strong fragrance, and high essential oil yield, this species possesses significant economic importance. Its petal-derived essential oil, often referred to as “liquid gold” is used in perfume, cosmetics, aromatherapy, spices, and nutrition industry [27]. Furthermore, *R. rugosa* is rich in valuable secondary metabolites, including flavonoids, gallic acid derivatives, and polysaccharides, which are extensively utilized in pharmaceutical applications [28,29]. In 1967, Hill successfully induced callus from stem tissue of hybrid tea rose, although the culture subsequently generated only shoot primordia [30]. In the following decades, researchers focused extensively on achieving complete plant regeneration from various tissue and organ explants [31,32,33,34,35,36], yet the potential of non-embryogenic callus for studying plant genetic transformation systems remained largely overlooked. Here, our study aims to establish a genetic transformation system using non-embryogenic calli to provide an effective platform for validating rose secondary metabolism genes in future studies.

## 2. Materials and Methods

### 2.1. Plant Materials and Media

‘Bao white’ embryogenic and non-embryogenic calli were cultivated in a growth chamber under controlled conditions (22 °C, dark). The relevant media for proliferation, differentiation and genetic transformation are presented in Table 1. The physiological indicators of PRO and CAT were measured using assay kits (Jiancheng, Nanjing, China).

### 2.2. Vector Construction

Our previous study had screened the *RrPSY1* gene involved in carotene synthesis in rose fruit [37], so we constructed *RrPSY1* gene overexpression vector. The coding sequences of *RrPSY1* were cloned into the pFAST-R05 vector to construct the overexpression vectors 35S:RrPSY1-GFP. The specific primers for the *RrPSY1* gene are listed in Table S1.

### 2.3. Transformation Procedure

**Figure 1:**
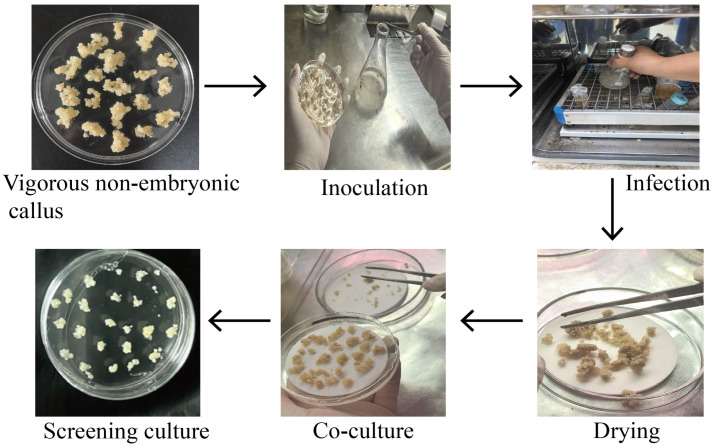
Agrobacterium tumefaciens-mediated transformation program of *R. rugosa* non-embryogenic callus.

Vigorous non-embryogenic calli that had been grown on proliferation medium for one month were selected for inoculation. The calli were immersed in an Agrobacterium infection solution harboring either the 35S:RrPSY1-GFP construct or the empty vector 35S:GFP (as a negative control) and incubated in shaker incubator at 28 °C and 200 rpm for about 1 h. Subsequently, they were briefly placed on dry, sterilized filter paper in a Petri dish to remove excess bacterial solution and prevent overgrowth during co-culture. The calli were then transferred onto a co-culture medium overlaid with sterile filter paper and incubated for 2–3 days. Finally, the calli were rinsed with sterile water, blotted dry, and transferred to screening medium (Figure 1).

### 2.4. Carotenoid Detection

Carotenoids were extracted following the reported method [38]. All the extraction and analysis were conducted in dim light to minimize photo-oxidative reactions. 2.0 g freeze-dried calli powder were homogenized in 20 mL of extraction buffer (water-saturated n-butanol) for 30 s by a vortex and kept shaking for 3 h at room temperature. Then, the mixture was centrifuged at 4000× *g* for 15 min at 4 °C, and the resulting supernatants were collected. The concentrations of total yellow pigment were determined by measuring the absorbance at wavelengths of 450 nm using an enzyme-labeled instrument (SP-Max2300A, Shanghai, China), with water-saturated n-butanol used as the control. Three technical replicates were performed for each sample. Concentrations were calculated according to the methods [39].

### 2.5. GFP Fluorescence Observation

The 35S:GFP and 35S:RrPSY1-GFP transgenic calli on the screening medium were identified by GFP fluorescence signal. GFP signal visualization and image acquisition using a stereo microscope (SMZ800N, Nikon, Japan). The fluorescence signal was visualized using 480 nm excitation light and 510 nm emission light.

### 2.6. PCR Identification of Positive Calli

Genomic DNA from WT, 35S:GFP and 35S:RrPSY1-GFP transgenic calli were isolated by the CTAB method [40]. The extracted DNA was subjected to Polymerase chain reaction (PCR) amplification with GFP-specific primers (Table S1) to detect the target fragment in the transgenic calli.

### 2.7. Statistical Analysis

Tukey’s honestly significant difference (HSD) tests were employed to determine statistical significance, when *p* < 0.05 the difference was defined as significant.

## 3. Results

### 3.1. Comparison of Embryogenic and Non-Embryogenic Callus

**Figure 2:**
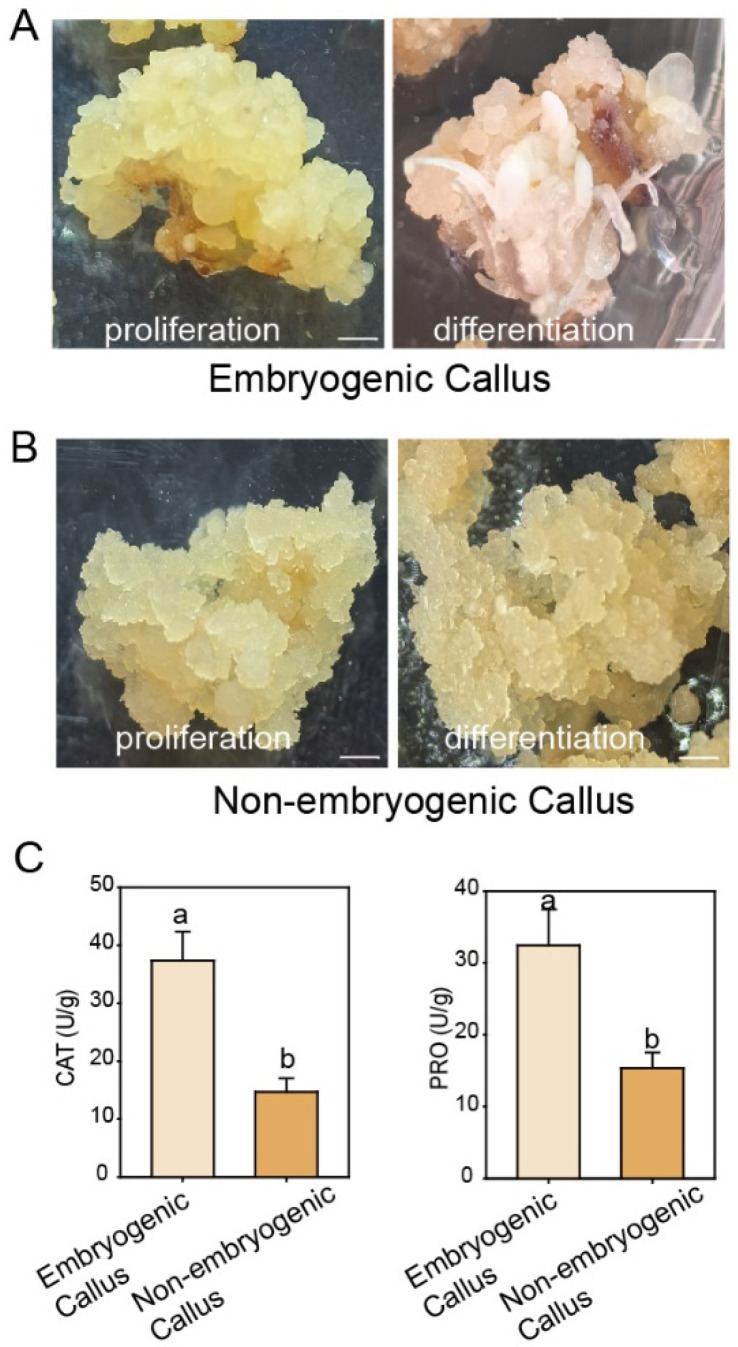
Comparison of embryogenic and non-embryogenic callus. Representative images of embryogenic callus (**A**) and non-embryogenic callus (**B**) growing on proliferation medium and differentiation medium. Bars = 1 mm. (**C**) CAT activity and PRO content of embryogenic callus and non-embryogenic callus tissues. Data were means of 3 biological replicates ± SD (*n* = 3). Different letters indicate significant differences as determined using Tukey’s HSD test (*p* < 0.05).

Observation of the morphological characteristics revealed distinct differences between embryogenic and non-embryogenic calli following a period of growth on proliferation medium. Embryogenic calli appeared as compact, yellow tissue masses, whereas non-embryogenic calli exhibited a watery and soft consistency. Upon transfer to differentiation medium, the embryogenic callus developed granular protrusions that gradually differentiated into new tissues, demonstrating its capacity for somatic embryogenesis. In contrast, the non-embryogenic callus remained watery and turned darker in color (Figure 2A,B). These morphological characteristics are consistent with the observations of other species [3,11].

We next measured the CAT activity and PRO content in the two callus types. These key physiological indicators reflect the tissues’ ability to scavenge reactive oxygen species, their level of metabolic activity, and their overall stress resistance. The results revealed that both CAT activity and PRO content were significantly higher in embryogenic callus than in non-embryogenic callus (Figure 2C), suggesting enhanced stress resistance in the embryogenic type, which may facilitate its subsequent differentiation.

**Figure 3:**
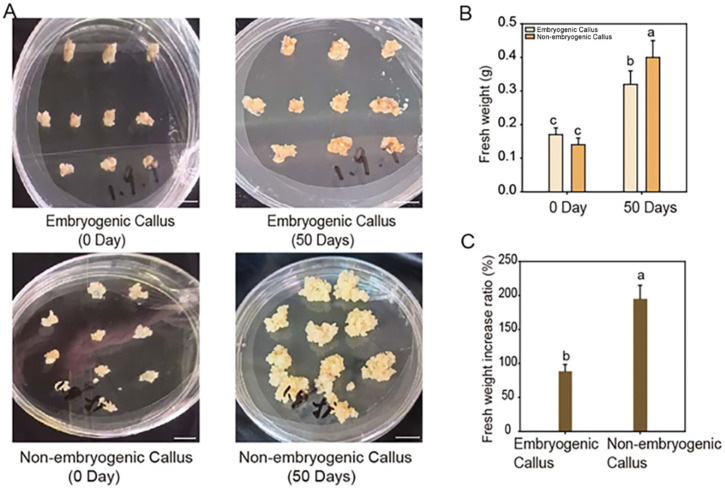
The non-embryogenic calli exhibited a higher growth rate compared to the embryogenic calli. (**A**) Morphology of the two types of calli before and after a 50-day culture period on proliferation medium. Bars = 10 mm. (**B**) Fresh weight and (**C**) growth rate of fresh weight of the calli before and after the 50-day culture period. Mean values ± SD were shown from 10 biological replicates (*n* = 10). Different letters indicate significant differences as determined using Tukey’s HSD test (*p* < 0.05).

Subsequently, we compared the growth rates of the two types of callus: embryogenic and non-embryogenic calli of similar initial size and growth status were, respectively, inoculated onto fresh proliferation medium. After 50 days of culture, the non-embryogenic callus exhibited a significantly higher growth rate than the embryogenic callus (Figure 3A,B). The fresh weight of embryogenic callus increased by approximately 100%, while that of non-embryogenic callus exhibited a markedly greater increase of nearly 200% (Figure 3C). These results suggest that non-embryogenic callus possesses a greater proliferative capacity.

### 3.2. Antibiotic Concentration Screening

**Figure 4:**
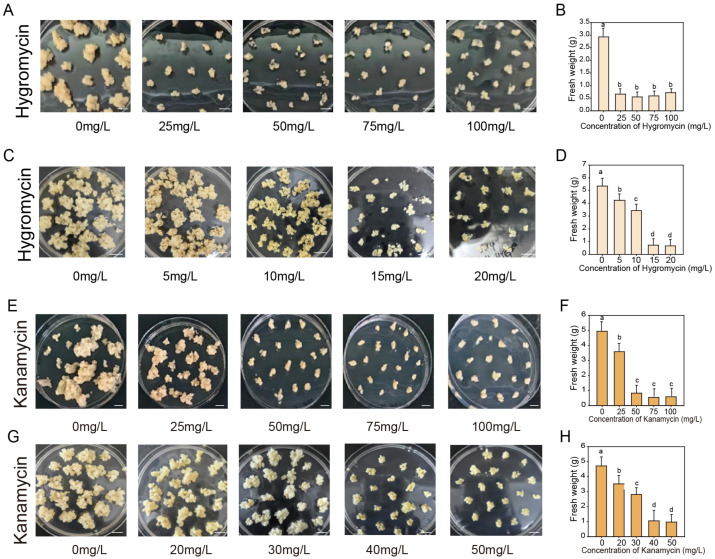
Effect of different concentrations of kanamycin and hygromycin on the proliferation of non-embryogenic calli. (**A**,**C**,**E**,**G**) Representative images show the growth state of non-embryogenic calli cultured for one month on proliferation medium containing varied concentrations of kanamycin and hygromycin. Bars = 10 mm. (**B**,**D**,**F**,**H**) Fresh weight of calli cultured at the antibiotic concentrations corresponding to panels (**A**,**C**,**E**,**G**). Mean values ± SD were shown from 3 biological replicates (*n* = 3). The different letters above the bars indicate significant differences as determined by Tukey’s HSD test (*p* < 0.05).

In the establishment of a genetic transformation system, the selection of appropriate and efficient marker genes is crucial for identifying positive transgenic materials. Kanamycin and hygromycin are widely used as screening agents in plant genetic transformation. To determine the optimal concentration of hygromycin and kanamycin for callus transformation, non-embryogenic calli of uniform size were cultured on proliferation medium supplemented with 0, 25, 50, 75, and 100 mg/L of hygromycin or kanamycin, and their growth status was assessed after one month. Both antibiotics significantly inhibited calli growth (Figure 4A,E). Complete inhibition of calli proliferation was observed at 25 mg/L for hygromycin or 50 mg/L for kanamycin (Figure 4A,B,E,F). Further refinement of antibiotic concentrations revealed that the minimal inhibitory concentration was 30–40 mg/L for kanamycin and 10–15 mg/L for hygromycin, respectively (Figure 4C,D,G,H). Based on these results, 35 mg/L kanamycin and 13 mg/L hygromycin were selected for use in subsequent experiments.

### 3.3. Genetic Transformation of Callus with RrPSY1 Gene

PSY is a key rate-limiting enzyme in the carotenoid biosynthesis pathway. Previous studies have demonstrated that overexpression of *PSY* in calli promotes carotenoid accumulation and results in an orange pigmentation, which serves as a visual marker for efficiently identifying positive transformants [41,42]. In *R. rugosa*, *RrPSY1* expression is significantly upregulated during fruit ripening, yet its function remains uncharacterized [37]. Therefore, we transferred the *RrPSY1* gene into the non-embryogenic callus using the method described in the materials and methods section.

**Figure 5:**
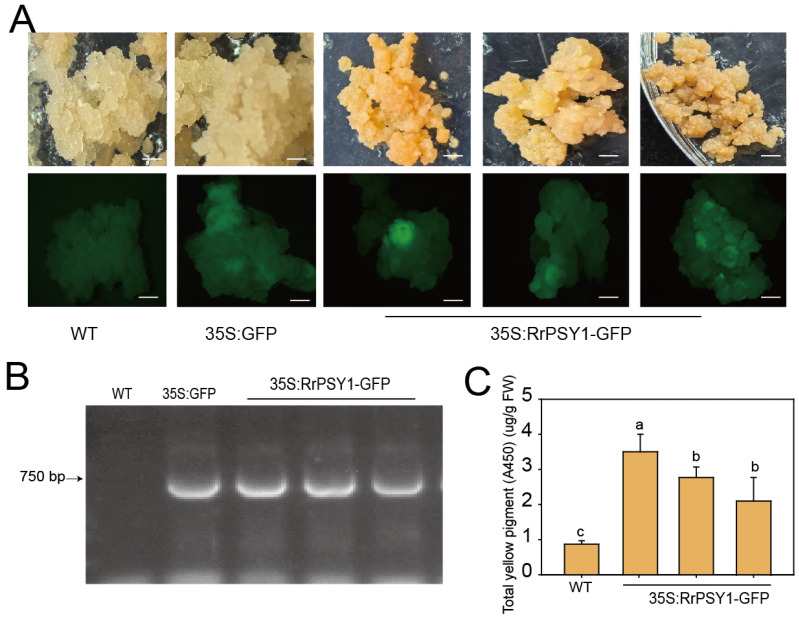
Identification of positive transgenic callus. (**A**) Color phenotype and GFP fluorescence in WT, 35S:GFP and 35S:RrPSY1-GFP transgenic calli. Bars = 20 mm. (**B**) PCR detection of GFP fragment in WT, 35S:GFP and 35S:RrPSY1-GFP transgenic calli. (**C**) Total yellow pigment (A450) in WT and 35S:RrPSY1-GFP transgenic calli. Mean values ± SD were shown from 3 technical replicates (*n* = 3). The different letters above the bars indicate significant differences as determined by Tukey’s HSD test (*p* < 0.05).

To identify successfully transformed calli, we first screened for GFP fluorescence. Approximately 6.96% of the 35S:RrPSY1-GFP transgenic calli exhibited GFP signals, which were also present in the 35S:GFP controls but were absent in the wild-type (WT) callus (Figure 5A). This result was further verified by PCR, which amplified the GFP fragment exclusively in the 35S:RrPSY1-GFP transgenic calli and the 35S:GFP controls (Figure 5B). Furthermore, quantification of total yellow pigment revealed a 3–4 fold increase in the 35S:RrPSY1-GFP transgenic calli compared to WT (Figure 5C). Together, these results confirm the successful overexpression of *RrPSY1* in transgenic calli, which consequently enhanced carotenoid accumulation.

## 4. Discussion

Over the years, genetic transformation system has been a key bottleneck in molecular biology. Serving as a fundamental bridge between genotype and phenotype, it has become an indispensable tool for functional genomics. With the progress of genome sequencing technologies, complete genome assemblies have been published for multiple Rosa species, including *R. chinensis*, *R. multiflora*, and *R. rugosa* [43,44,45]. These advancements have established a critical foundation for investigating key ornamental traits, enhancing stress resistance, and advancing molecular breeding programs in Rosa species. Currently reported genetic transformation systems for Rosa plants include *Agrobacterium*-mediated somatic genetic transformation, virus-induced gene silencing (VIGS) technology, transient transformation techniques and *Agrobacterium rhizogenes*-mediated hairy root technology [31,46,47,48,49]. The continuous refinement of these technologies has significantly accelerated the molecular breeding of Rosa species. In our study, we established a novel genetic transformation system using non-embryogenic callus, providing new technical support for molecular biology research in Rosa genus plants.

The study demonstrated that stress factors can promote the initiation and development of somatic embryos. In our research, we observed that the CAT activity and PRO content were significantly higher in embryogenic callus than in non-embryogenic callus (Figure 2C), suggesting enhanced stress resistance in embryogenic callus. Consistent with reports in other plants, the antioxidant enzyme system is activated in embryogenic callus, leading to the accumulation of antioxidant enzymes that scavenge stress-induced reactive oxygen species (ROS) and mitigate oxidative damage [50,51,52]. The observed physiological differences may be linked to the developmental potential for whole-plant regeneration. Unlike embryogenic callus, non-embryogenic callus does not invest energy in maintaining regenerative capacity or sustaining high-level stress protection. Instead, it can redirect metabolic resources toward rapid growth and proliferation (Figure 3).

Appropriate and efficient marker genes are essential for monitoring transformation processes and screening positive transgenic lines. In this study, we performed antibiotic concentration screening on non-embryogenic callus using a range of concentration gradients. Our results identified effective selection concentrations of 30–40 mg/L for kanamycin and 10–15 mg/L for hygromycin (Figure 4). In contrast, a sensitivity threshold of 75 mg/L for kanamycin was reported for embryogenic callus in rose [36]. This difference may be related to the genotypes, metabolic efficiency and stress resistance of the two calli. In Tamarillo, primary metabolite analysis revealed that non-embryonic callus exhibits significantly higher levels of carbohydrate metabolism, glycolytic activity, and amino acid metabolism than embryonic callus [3]. In our study, the embryogenic callus exhibited higher PRO content and CAT activity. These advantageous physiological traits may have collectively enhanced the tolerance of the embryogenic callus to external stresses.

In fruit trees, callus-based genetic transformation systems have been widely employed for functional validation of genes associated with anthocyanin biosynthesis, fruit peel coloration, and the metabolism of secondary compounds such as flavonoids and carotenoids. Nevertheless, the application of callus-based systems for gene function validation in ornamental flowers remains limited. To date, only Jiang et al. (2023) have demonstrated that overexpression of *RhWRKY33a* or *RhPLATZ9* in rose calli significantly reduces ROS accumulation [53]. This scarcity of studies may be attributed to the currently underdeveloped genetic transformation system in ornamental flowers plants. In this study, the *RrPSY1* gene was transformed into non-embryogenic calli. Positive transgenic calli were identified based on GFP fluorescence and confirmed by PCR. Carotenoid content analysis showed that the *RrPSY1* overexpression lines accumulated significantly higher levels of carotenoids compared to the WT (Figure 5). These results demonstrate that this system can be effectively used for functional validation of genes.

## 5. Conclusions

In this study, we conducted the first comparative analysis of the morphological and physiological characteristics between embryogenic and non-embryogenic calli in *R. rugosa*.

The non-embryogenic callus was characterized as a soft, water-soaked tissue exhibiting relatively rapid growth but poor stress resistance. Antibiotic sensitivity assays identified the optimal selection concentrations for non-embryogenic callus as 35 mg/L for kanamycin and 13 mg/L for hygromycin. Subsequently, using *Agrobacterium*-mediated transformation of the *RrPSY1* gene, we successfully established a stable genetic transformation system for non-embryogenic callus. This system provides a useful technical platform for the manipulation of metabolic products and the verification of related gene functions in rose.

## Figures and Tables

**Table 1:** Media used for calli transformation.

Medium Type	Composition
proliferation medium	MS + 1 mg/L 2, 4-D + 0.01 mg/L 6-BA + 3 g/L gel + 40 g/L sucrose
differentiation medium	1/2 MS + 1 mg/L 6-BA + 3 g/L gel + 30 g/L sucrose
Agrobacterium infestation	1/2 MS + 30 g/L Glucose + 100 mg/L AS
co-culture medium	MS + 40 g/L Glucose + 1 mg/L 2, 4-D + 0.01 mg/L 6-BA + 3 g/L gel + 200 μmol AS
screening medium	MS + 45 g/L Glucose + 1 mg/L 2, 4-D + 0.01 mg/L 6-BA + 3 g/L gel + 300 mg/L cef + 35 mg/L Kana

## Data Availability

The original contributions presented in this study are included in the article/Supplementary Material. Further inquiries can be directed to the corresponding author.

## Supplementary Materials

The following supporting information can be downloaded at https://www.mdpi.com/article/10.3390/cimb47110894/s1.
